# Rewinding the molecular clock in the genus *Carabus* (Coleoptera: Carabidae) in light of fossil evidence and the Gondwana split: A reanalysis

**DOI:** 10.1371/journal.pone.0256679

**Published:** 2021-09-22

**Authors:** Lars Opgenoorth, Sylvia Hofmann, Joachim Schmidt

**Affiliations:** 1 Faculty of Biology, Plant Ecology and Geobotany, Philipps-University Marburg, Marburg, Germany; 2 WSL Swiss Federal Research Institute, Birmensdorf, Switzerland; 3 Centre of Taxonomy and Evolutionary Research, Zoological Research Museum Alexander Koenig, Bonn, Germany; 4 Institute of Biosciences, General and Systematic Zoology, University of Rostock, Rostock, Germany; Nanjing Agricultural University, CHINA

## Abstract

Molecular clocks have become powerful tools given increasing sequencing and fossil resources. However, calibration analyses outcomes depend on the choice of priors. Here, we revisited the seminal dating study published by Andújar and coworkers of the genus *Carabus* proposing that prior choices need re-evaluation. We hypothesized that reflecting fossil evidence and the Gondwanan split properly significantly rewinds the molecular clock. We re-used the dataset including five mitochondrial and four nuclear DNA fragments with a total length of 7888 nt. Fossil evidence for Oligocene occurrence of *Calosoma* was considered. Root age was set based on the fossil evidence of Harpalinae ground beetles in the Upper Cretaceous. Paleogene divergence of the outgroup taxa Ceroglossini and Pamborini is introduced as a new prior based on current paleontological and geological literature. The ultrametric time-calibrated tree of the extended nd5 dataset resulted in a median TMRCA *Carabus* of 53.92 Ma (HPD 95% 45.01–63.18 Ma), roughly 30 Ma older than in the Andújar study. The splits among *C*. *rugosus* and *C*. *morbillosus* (A), *C*. *riffensis* from the European *Mesocarabus* (B), and *Eurycarabus* and *Nesaeocarabus* (C) were dated to 17.58 (12.87–22.85), 24.14 (18.02–30.58), and 21.6 (16.44–27.43) Ma. They were decidedly older than those previously reported (7.48, 10.93, and 9.51 Ma). These changes were driven almost entirely by constraining the Carabidae time-tree root with a Harpalinae amber fossil at ~99 Ma. Utilizing the nd5 dating results of three well-supported *Carabus* clades as secondary calibration points for the complete MIT-NUC dataset led to a TMRCA of *Carabus* of 44.72 (37.54–52.22) Ma, compared with 25.16 Ma (18.41–33.04 Ma) in the previous study. Considering fossil evidence for Oligocene *Calosoma* and Late Cretaceous Harpalini together with the Gondwanan split as a new prior, our new approach supports the origin of genus *Carabus* in the Eocene. Our results are preliminary because of the heavy reliance on the nd5 gene, and thus will have to be tested with a sufficient set of nuclear markers. Additionally, uncertainties due to dating root age of the tree based on a single fossil and outgroup taxon affect the results. Improvement of the fossil database, particularly in the supertribe Carabitae, is needed to reduce these uncertainties in dating *Carabus* phylogeny.

## Introduction

The molecular clock has become an increasingly powerful tool in biogeography and phylogenetics owing to ever-increasing genomic and fossil calibration data [1]. However, phylogenetic dating is often performed in Bayesian frameworks, where the choice and number of calibration priors have a deciding impact on the dating results [1, 2]. Consequently, there is often a huge dating variance among studies, even those dealing with identical taxa and employing identical calibration points [3–5]. Important factors for these variances are the placement of fossils in a given phylogeny and the handling of geological priors. The first factor is a matter of taxonomic discussion among species group specialists. Recent methodological improvements for better analyses of hidden characters in fossils have included X-ray micro-computed tomography of amber inclusions to determine internal genital characteristics of tiny beetles [6, 7]. These improvements may help to resolve ambiguities in the long term. However, the handling of geological priors is a broader discussion, where the improvement can and should be somewhat more predictable and transparent across taxonomic groups. However, the almost-arbitrary choice of geological sources and thus, setting of respective molecular clocks is evident [8]. One classical geological event that has broadly left its imprint on biogeographic patterns is the split and fragmentation of the Gondwanan landmasses [9]. Studying the widely-reviewed biogeographical literature dealing with the Gondwanan split reveals that two general patterns emerge. Taxa, which are good dispersers and occur in a broad range of terrestrial habitats, have very diverse phylogeographic histories, often independent of the timing of the Gondwanan fragmentation. The evolutionary history of taxa with poor dispersal capabilities and often very specific habitat preferences on the other side, often reflect the trademark vicariance pattern. Examples range all the way from chironomid midges [10], stoneflies [11], scorpions [12], and anurans [13] to plants such as *Nothofagus* [14], but see [15]. Therefore, the geological record should be used to calibrate the molecular clock only for taxa which are poor dispersers.

Here, we revisit a seminal study on the calibration of the phylogeny of *Carabus* ground beetles [16] reflecting both the fossil evidence for the outgroup and recent geological as well as biogeographical consensus on the fragmentation of the Gondwanan landmasses. *Carabus* is generally described as a Holarctic genus that currently comprises approximately 940 species classified into 91 subgenera [17]. Its distribution includes Eurasia, Japan, Iceland, the Canary Islands, North Africa, and North America [17–21]. *Carabus* represents the most species-diverse terminal clade of the supertribe Carabitae, which also includes the Holarctic Cychrini, Andean Ceroglossini (= *Ceroglossus*), Australasian Pamborini (= *Pamborus* + *Maoripamborus*), and cosmopolitan Calosomina (= *Calosoma sensu lato*). The latter was identified as the sister group of *Carabus* based on molecular data [16, 22–25]that agrees with the morphological data [26].

Aside from the Messinian fossil of *Carabus cancellatus* sensu lato [27] and Late Chattian fossils of *Calosoma* [28], no additional fossil evidence can be conclusively assigned to Carabitae which is older than the Pliocene and Quaternary periods. However, this poor fossil evidence certainly does not reflect the evolutionary age of the group. Based on the evolutionary model proposed by Erwin in 1979 [29], Carabitae represents a very old lineage of Geadephaga, with its primary diversification reflecting continental drift events during the late Early Cretaceous. Penev et al. [26] proposed that species belonging to the genera *Calosoma* and *Carabus* were present at least in the early Cenozoic era. The dates of the molecular phylogenetic study of Toussaint & Gillet [25] correspond with these hypotheses. They estimated the origin of Carabitae to approximately 170 Ma, and that of the *Calosoma-Carabus* split in the Cretaceous. To estimate the divergence time, the split of Trachypachidae and Carabidae (estimated as 200 Ma) was used for the reanalysis of the data of a previous study [30] using 34 Carabitae outgroup fossils [31]. The most recent comprehensive analysis of Coleoptera molecular evolution done by McKenna et al. [32] showed that the Trachypachidae-Carabidae split is at 170 Ma and the *Calosoma-Carabus* split is in the Late Eocene and later, as estimated by Toussaint & Gillet [25]. To estimate the divergence time, McKenna et al. [32] selected 18 Carabitae outgroup fossils.

All these hypotheses contrast sharply with the molecular evolutionary models proposed by Andújar et al. [16] and Deuve et al. [17] that estimated divergence time based mainly on geological events. Deuve et al. proposed the first diversification of Carabitae in the Paleocene–Eocene, with a split of *Calosoma* and *Carabus* not occurring until prior to the Oligocene. The Oligocene–Miocene emergence of the megadiverse genus *Carabus* is surprising considering the fossil evidence in the Carabidae family. Recent studies of Baltic amber inclusions have clarified that representatives of modern ground beetle genera, even those from the subfamily Harpalinae, already existed during the Eocene along with certain fossil species of the extant genera *Calathus* of the tribe Sphodrini [33], *Coptodera* of Lebiini [34], and *Limodromus* of Platynini [35]. In addition, the presence of Harpalinae is evident in the fossil record since the early Late Cretaceous [36–38]. Finally, molecular genetic studies have demonstrated that Carabitae are phylogenetically older than Harpalinae [39–41], and that Harpalinae underwent rapid speciation during the Late Cretaceous and Early Cenozoic [36, 42, 43]. In this regard, the question arises on why Carabitae would have undergone this markedly long phylogenetic standstill of not less than 50–60 Ma, according to the timing proposed by Andújar et al. [16].

This dilemma led us to revisit the dating background of the molecular study of the genus *Carabus* by Andújar et al. [16]. As detailed in the Materials and Methods section, Andújar et al. [16] included several classical geological calibration events. These included the emergence of the Canary Islands, the opening of the Strait of Gibraltar, and disconnection events of Japan from the Asian mainland. In all three cases, we argue that their chosen approach is not plausible from palaeogeographical and palaeoecological standpoints, but rather reflects a common oversimplification of historical dispersal mechanisms. In addition, we focus on another important issue of the Carabitae evolution–from a biogeographical point of view, the South American genus *Ceroglossus* and the Australasian genera *Pamborus* and *Maoripamborus* are particularly remarkable and together, they form the sister clade to *Carabus* and Calosomina based on molecular data [24, 25]. A previous morphology-based hypothesis posited that the close relationship of *Pamborus* and *Maoripamborus* with the Cychrini tribe was based on convergence [44]. This view has also been confirmed by molecular data, since Cychrini were placed in the basal position within the Carabitae in previous studies [16, 25, 40]. The split of the South American and Australasian taxa offers an additional possibility to calibrate the *Carabus* phylogeny. Since Andújar et al. [16] neglected this calibration point, we propose that their time-tree massively underestimates the true age of the genus *Carabus*, as was already presumed by Toussaint & Gillet [25].

In light of these considerations, we hypothesize that i) Adding a root age based on fossil evidence for Harpalinae and the inclusion of the Gondwanan split will push the dating of the crown age of *Carabus* to at least the Eocene, and ii) A proper adjustment of the geological calibration points used by Andújar et al. [16] will resolve putative contradictions between those points and the fossil data, as well as the Gondwanan split. To test these hypotheses, we reanalyzed the datasets of Andújar et al. [16]. Specifically, our new calibration strategy was based on a review of recent geological and biogeographical literature dealing with i) Taxa that have a Gondwanan distribution, extracting minimum and maximum calibration ages, ii) The onset of the Canary Hotspot using the taxonomic split between mainland and island taxa instead of island taxa only, iii) The significant surface uplift of the Japanese island arc, and iv) The Miocene orogenesis in the western Mediterranean region. Finally, our findings of the *Carabus* time-tree are used to discuss the general need for a more differentiated and transparent usage of geological calibration points depending on life-history traits and habitat requirements of the taxa under study. Unlike the study of Andújar [16] that was especially concerned in deriving the evolutionary rates, we explicitly focused on the age of the genus *Carabus*, and not on reconstructing evolutionary rates.

## Methods

### Phylogenetic data sets

To ensure a direct comparability of dating results, we utilized three phylogenetic datasets presented by Andújar et al. [16], which was based on the alignments deposited by the authors at TreeBASE under the submission number 12410 (http://purl.org/phylo/tree-base/phylows/study/TB2:S12410).

The first dataset is based on nd5 sequences from 58 Carabidae species and is designated as the extended nd5 dataset. It includes 57 species of the supertribe Carabitae, with 51 species of the genus *Carabus*, representing 16 subgenera and 7 out of the 13 main *Carabus* clades identified by Deuve et al. [17], one species each from the genera *Calosoma*, *Ceroglossus*, *Maoripamborus*, and *Cychrus*, two species of *Pamborus*, and the Harpalinae species *Abax parallelepipedus* as the outgroup taxon.

The second dataset includes 34 specimens of the Carabidae family. There are 19 species of the genus *Carabus*, two species of its sister taxon *Calosoma*, one species each of *Ceroglossus* and *Cychrus* (representatives of the supertribe Carabitae), the Nebriitae species *Leistus spinibarbis*, and Harpalinae species *Laemostenus terricola* as outgroup taxa. Alignments are available for the mitochondrial genes *cox1-A*, *cox1-B*, *nd5*, *cytb*, *rrnL*, and the nuclear genes *LSU-A*, *LSU-B*, *ITS2*, and *HUWE1*.

The third dataset is a subset of the second dataset. It includes only the 19 *Carabus* species making up theingroup.

### Calibration strategy

The calibration scheme of Andújar et al. [16] used on the nd5 extended dataset is shown in Table 1. Table 2 shows the equivalent calibration scheme used in this study. Specifically, we followed the approach of the aforementioned authors only concerning the *Carabus cancellatus* fossil from France (F1). Our treatment of the other calibration priors will be explained in the following paragraphs. Similar to Andújar et al. [16] we used the resulting calibrated phylogeny to obtain ages for three well-supported cladogenetic events in the phylogeny of *Carabus*, and included the identical nomenclature for the respective splits between *Carabus* (*Macrothorax*) *rugosus* and *C*. (*Macrothorax*) *morbillosus* (Node A), *C*. (*Mesocarabus*) *riffensis* from the European *Mesocarabus* clade (Node B), and the split between the sister subgenera *Eurycarabus* and *Nesaeocarabus* (Node C). These nodes were selected by Andújar et al. [16] because they are not affected by systematic conflicts, are old enough to avoid time dependence effects, and not excessively affected by saturation of molecular changes. We used TreeStat 1.7.1 from the BEAST package to recover node ages from the sample of the MCMC search in BEAST and used the R MASS package [45] to obtain the gamma function using the “fitdistr” function (Table 4).

**Table 1:** Calibration scheme from Andújar et al. 2012 [16].

Evolutionary event: Node	Calibration event	Event age (Ma)	Priors on node ages	Prior 95% age interval
**C | Split between the Canarian endemics *Carabus* (*Nesaeocarabus*) *coarctatus* and *C*. (*N*.) *abbreviatus***	Volcanic emergence of Gran Canaria	14.5	Uniform (a = 0, b = 14.5)	0.03–14.14
**F *| Carabus* (*Tachypus*) *cancellatus* fossil**	Messinian deposits of Cantal (France)	5	Lognormal (μ = 25, σ = 1.5, offset = 5)	5.4–158.5
**J1 | Radiation of *Damaster***	Final disconnection of Japan from mainland	3.5	Truncated Normal (μ = 3.5, σ = 1, a = 0.1, b = 1000)	1.55–5.46
**J2 | Radiation of *Leptocarabus***	Final disconnection of Japan from mainland	3.5	Truncated Normal (μ = 3.5, σ = 1, a = 0.1, b = 1000)	1.55–5.46
**J3 | Radiation of *Ohomopterus***	Final disconnection of Japan from mainland	3.5	Truncated Normal (μ = 3.5, σ = 1, a = 0.1, b = 1000)	1.55–5.46
**J4 | Split between subgenus *Isio-carabus* and *Ohomopterus***	Initial disconnection of Japan from mainland	15	Normal (μ = 15, σ = 1)	13.04–16.96
**M2 | Split between *Carabus* (*Eurycarabus*) *genei* from Corsica and North African *Eurycarabus***	Opening Gibraltar Strait	5.33	Exponential (μ = 0.5, offset = 5.3)	5.31–7.14
**M3 | Split between two *Carabus* (*Rhabdotocarabus*) *melancholicus* subspecies**	Opening Gibraltar Strait	5.33	Exponential (μ = 0.5, offset = 5.3)	5.31–7.14

Denomination of the evolutionary events as stated there.

**Table 2:** Calibration strategy of this study.

Evolutionary event: Node	Calibration event	Event age (Ma)	Priors on node ages	Prior 95% age interval
**C1 |** Split between two Canarian endemic species: *Carabus* (*Nesaeocarabus*) *coarctatus* and *C*. (*N*.) *abbreviatus*	Volcanic emergence of Tenerife	11.9	Log-normal (μ = 16.7, σ = .82, offset = 0)	2.39–59.5
**C2 |** Split between the Canarian endemic *Nesaeocarabus* from the mainland *Eurycarabus*	Volcanic emergence of the Canary Hotspot	60	Uniform (lower = 0, upper = 60, offset = 0)	1.5–58.5
**F1 |***Carabus* (*Tachypus*) *cancellatus* fossil	Messinian deposits of Cantal (France)	5.3	Log-normal (μ = 25, σ = 1.5, offset = 5)	5.43–163.5
**F2 |***Calosoma agassizi* fossil	*Carabus*—*Calosoma* split. Minimum age for *Calosoma*	23	Uniform (lower 23, upper = 160, offset = 0)	26.5–160
**GO |** Split of Pamborini and Ceroglossini	Split of Australia between Antarctica/South America	32–50	Log-normal (μ = 78, σ = 0.42, offset = 0)	32.1–163.5
**J1 | Radiation of *Damaster***	Marked relief emergence	15	Uniform (lower = 0, upper = 15)	0.375–14.3
**J2 | Radiation of *Leptocarabus***	Marked relief emergence	15	Uniform (lower = 0, upper = 15)	0.375–14.3
**J3 | Radiation of *Ohomopterus***	Marked relief emergence	15	Uniform (lower = 0, upper = 15)	0.375–14.3
**M2 |** Split between *Carabus* (*Eurycarabus*) *genei* from Corsica and North African *Eurycarabus*	Surface uplift of the North African mountain ranges	15	Uniform (lower = 5.5, upper = 15, offset = 0)	5.54–14.8
**M3 |** Split between two *Carabus* (*Rhabdotocarabus*) *melancholicus* subspecies	Surface uplift of the North African mountain ranges	15	Uniform (lower = 5.5, upper = 15, offset = 0)	5.54–14.8
**RO |** Root	Fossil Harpalinae in Burmese amber	99	Log-normal (μ = 130, σ = 4.25, offset = 98.17)	98.2–163.5

Denomination of the evolutionary events follows the study by Andújar et al. [16] for better comparability.

### Canary Islands prior

Using endemic lineages from island hotspots to date phylogenetic trees remains a problematic approach because the true age of a lineage might be older than the islands themselves, given their hotspot origin (see [46] for a comprehensive discussion of this problem). Andújar et al. [16] used the emergence of Gran Canaria (approximately 14.5 Ma) to date the maximum age of the split of *Carabus* (*Nesaeocarabus*) *coarctatus* (Gran Canaria) and *C*. (*N*.) *abbreviatus* (Tenerife). It is very probable that this split occurred along with the emergence of the younger Tenerife (11.9 Ma) [47]. However, it is not unlikely that the split of these species occurred on one of the presumed older islands that are submerged today. In this scenario, the migration of *Nesaeocarabus* to Gran Canaria and Tenerife was possible from any older island. The date of their respective submergence is unknown. Consequently, using the emergence of only one of the recent islands to date the maximum age of the split within *Nesaeocarabus* is potentially misleading and needs to be relaxed to account for the alternative possibility. This is achieved using the age of the Canary Hotspot (60 Ma based on kinematic studies; [48] as the maximum age of crown *Nesaeocarabus* and a log-normal distribution with a maximum probability of 11.9 Ma.

Because, theoretically, the split of *Nesaeocarabus* from mainland *Eurycarabus* was possible together with the upstart of the Canary Hotspot, we additionally used the age of the hotspot as the maximum age of stem *Nesaeocarabus* and a uniform distribution, as no additional information is available that would favor a specific time.

### Japan prior

The geological and geographical evolution of the Japanese island arc system is highly complex and not fully understood. This necessitates particular caution when using geological events to calibrate the molecular clock in deep times in species groups that are adapted to the temperate climate but for which the dispersal power is unknown. For example, Andújar et al. [16] used the initial disconnection of Japan from the Asian mainland at approximately 15 Ma to time-calibrate the split of the Japanese endemic *Ohomopterus* from mainland *Carabus* lineages. For the same split, Nagata et al. [49] used geological evidence of 3–1.7 Ma for the final disconnection of Japan to date the phylogenetic tree in *Ohomopterus*. Consequently, a very recent origin of the terminal lineages was estimated, in significant contrast to the results of Andújar et al. [16].

It is difficult to associate the evolution of stem *Ohomopterus* with any particular event in the geological and geographical evolution of the Japanese island arc. On one hand, Far East Asia with its dense ensemble of complex orogenetic systems of Kamchatka, Sikhote Alin, Sakhalin, and Korean peninsulas, the Japanese and Kurile island arcs, was geomorphologically diverse long before the initial disconnection of Japan [50, 51]. During the Late Cenozoic, the occurrence of suitable habitats for temperate *Carabus* has to be assumed, particularly along slopes of the many more-or-less separated mountains of the area. Consequently, it is likely that separation of the lineages was linked to the particular geomorphology of the area and the resulting differences in the regional and local climates, and has thus significantly predated the splitting events of the Japanese islands from continental Asia.

On the other hand, the presence of fully developed hindwings cannot be excluded for the ancestors of this group and other groups of *Carabus* ground beetles. This is because most of the main *Carabus* lineages were able to fly at least in the evolutionary history of the respective lineages, as evidenced by their phylogeny [17]. Colonization of the Japanese islands by *Ohomopterus*, and by *Damaster* and *Leptocarabus* beetles, was not necessarily connected with the presence of land bridges. Thus, the colonization was possible at any time following the terrestrial evolution of the island arc, provided that suitable habitats were present. This view is also supported by the phylogeny presented by Andújar et al. [16]. Based on these results, the split of Japanese endemic *Damaster* from the East Asian mainland *Acoptolabrus*, as well as the split of the Japanese endemic *Leptocarabus* clade from Korean representatives of that subgenus occurred at different times in the Late Miocene and significantly after the evolution of stem *Ohomopterus*. We suggest that there is no logical reason to hypothesize the initial disconnection of Japan from the Asian mainland as a prior on node age of any of these taxa.

The terrestrial evolution in some parts of present-day Japan began in approximately 24–23 Ma [52, 53]. However, there is no information regarding the ecological conditions in the area at that time. Starting from 15 Ma, intensive volcanic activity caused by the subduction of the Philippine Plate formed chains of volcanoes that were active until the Plio-Pleistocene and were subsequently replaced by modern volcanic chains starting from 7 Ma [52]. Thus, a marked relief and diverse local climatic conditions, including those suitable for temperate *Carabus* beetles, must be assumed to have been continuously present in the area of present-day Japan since at least the Mid Miocene. Most likely, ongoing intensive orogenesis and the resulting spatiotemporal diversity of the ecological conditions were the main drivers of the radiation within the *Ohomopterus*, *Damaster*, and *Leptocarabus* ground beetle lineages, rather than disconnection events of Japan from the mainland.

As a consequence, we changed the dating strategy of Andújar et al. [16] in several ways. First, we omitted the ‘initial disconnection of Japan from mainland’ calibration point because of the high probability that this event did not impact the *Carabus* evolution. Second, we set 15 Ma as the maximum age for the basal splits within the Japanese endemic *Ohomopterus*, *Damaster*, and *Leptocarabus* clades. This was done with the assumption that the beginning of intensive orogenesis was a possible evolutionary driver. We used a uniform distribution, as no additional information is available that would favor a specific time.

### Neogene orogenesis and geographical development in the Western Mediterranean region

During the Neogene period, Europe and its immediately adjacent regions underwent marked changes in their main geological and geographical features, with complex reorganizations of the terrestrial environments in the Mediterranean region [54]. Until the Pliocene, the climate was significantly warmer than it is today [55]. Tropical-subtropical environments have to be assumed for the lowland areas of North Africa and the southern parts of Europe. During the Messinian, the Mediterranean Basins were hot and dry, with spacious salt marshes present, and warm temperate conditions and mesophilic forests developed along slopes of the mountain belts [56, 57]. Mountains with various suitable habitats for temperate *Carabus* had been uplifted much earlier on both sides of present-day Gibraltar Strait [58, 59]. In the Betic-Rif system, rapid exhumation is assumed to have occurred during the Late Oligocene–Early Miocene (27–18 Ma; [60, 61]). In the Maghrebian, extensional deformation probably occurred at 25–16 Ma [62]. These mountain ranges became sufficiently high in the Mid Miocene. In addition, the uplift of the immediately adjacent Atlas Mountains has been attributed to Cenozoic thickening of the crust and Middle to Late Miocene thinning of the mantle lithosphere related to a shallow mantle plume [63, 64]. An appreciable part of the paleo-elevations has been attributed to this latter mechanism [64]. Thus, we conclude that sufficient heights for suitable *Carabus* habitats developed south of present-day Gibraltar Strait since at least 15 Ma.

As previously discussed for the Japan prior, in the western Mediterranean region, separation of the lineages was probably linked to Neogene orogenesis and the resulting differences in regional and local climates. Splitting events in *Eurycarabus* and *Rhapdotocarabus* beetles may have predated the post-Messinian opening of the Gibraltar Strait markedly. Consequently, the age of the Neogene surface uplift in the western Mediterranean region on both sides of the Gibraltar Strait has to be considered as the maximum possible age of crown *Eurycarabus* and *Rhapdotocarabus*. In this respect, we deviate from the calibration strategy used by Andújar et al. [16], who used an exponential distribution and accepted a prior on the node age of a maximum of 7.14 Ma within their 95% age interval. Instead, we have used a uniform distribution, since no conclusive information favoring a specific time is available.

### Split of Australia from Antarctica/South America

The breakup of Gondwana has been reflected in several ways in biogeographic and phylogeographic reconstructions. For example, Upchurch [65] proposed four broadly different models: The Samafrica model, Africa-first model, Pan-Gondwana model, and trans-oceanic dispersal. However, we are presently interested only in the split of Australia from Antarctica or South America to reflect the split between *Pamborus* with its Australian distribution and *Maoripamborus* with its New Zealand distribution (that together form the tribe Pamborini) from *Ceroglossus* (that forms the monotypic tribe Ceroglossini) with its South American distribution. Numerous studies have found reticulate histories along with the split of Australia between the Late Cretaceous and the Eocene. At this time, a shallow seaway between Australia and Antarctica was likely the last land passage between these continents [13]. Recently, it was proposed that this final split was a diachronous seafloor spreading that started in the West from 93–87 Ma, progressed to central Great Australia from 85–83 Ma, was followed by separation in the western Bight at ~65 Ma, and finalized in the Terre Adelie-Otway region at approximately 50 Ma (summarized in [66]). These authors discussed that there is still considerable uncertainty concerning the history of this breakup. The authors proposed that the oldest confident interpretations of magnetic seafloor anomalies date to approximately 45 Ma, when Australia and Antarctica finally drifted apart [66]. However, the South Tasman Rise was already submerged as deeply as 1000 m between 50 and 32 Ma [67] making passage difficult for beetles. In summary, we implemented a conservative dating approach concerning *Carabus* by choosing a calibration age with a minimum split at 32 Ma. Due to new geological evidence [68], we set the maximum age to 163.5 Ma instead of 159 Ma, as in Andújar et al. [16] and Deuve et al. [17]. This date is attributed to the oldest fossils, which definitely belong to the Carabidae, and were described from the Karabastau series of the Upper Jurassic of Kazakhstan [38]. Because the precise age of this series was not given, the currently accepted date of the Middle Jurassic/Upper Jurassic boundary has been used as the maximum age. The much-older fossil *Lithorabus incertus* from the Lower Jurassic of Kyrgyzstan was also described within the Carabidae family [38]. However, this was based on a very poor imprint of a few parts of the exoskeleton, making the systematic assignment doubtful. We chose a log-normal distribution (see Table 2).

### Fossil evidence

Consistent with Andújar et al. [16], we assigned crown *Tachypus* a log-normal distribution with a minimum age of 5.3 Ma based on a Messinian fossil in deposits of Chantal, France, and a maximum age of 163.5 Ma following the same logic as described in 3.2.4. We additionally assigned stem *Calosoma* a uniform distribution with a minimum age of 23 Ma based on fossil evidence of *Calosoma* in upper Oligocene deposits of Aix-en-Provence [28, 69], again with a maximum age of 163.5 Ma.

Andújar et al. [16] used *Abax parallelepipedus* as the Carabitae outgroup taxon for their nd5 data analyses. The genus *Abax* is representative of the Pterostichini tribe of the ground beetle subfamily Harpalinae; this genus includes the most number of species in Carabidae [43]. Morphological and molecular genetic phylogenies have consistently indicated the terminal position of Harpalinae within Carabidae [39, 40, 70]. Harpalinae fossils have been described from Upper Cretaceous deposits in South Kazakhstan, Beleutin formation, Turonian (93.5–89.0 Ma, [38], and from Burmese amber (ca. 99 Ma, R. G. Beutel et al., 2020; Liu et al., 2015). Therefore, we set the prior for Harpalinae to 99 Ma (see Table 2), with a maximum age of 163.5 Ma.

### Calibration analyses with an assessment of the input of each calibration point

All phylogenetic analyses were conducted with BEAST2 [71] and run on the CIPRES Cyberinfrastructure for Phylogenetic Research [72]. To assess the relative contribution of each calibration point of the respective priors, we ran seven analyses. In these analyses, one of the individual calibration points from Table 2 was removed in each of the runs, respectively, with one run including all priors. The impact of individual calibration points was assessed using violin plots produced with the R package vioplot [73]. Since the root prior proved to have a decisive input on the overall dating, a second violin plot was drawn that excluded the root calibration in all other stepwise omissions. In all approaches, *Calosoma* was constrained as the sister clade to *Carabus* and used *Abax* as an outgroup, since these relationships have been well established [40, 70]. We additionally constrained *Carabus* (*Limnocarabus*) *clatratus* as the most basal split within the nd5 *Carabus* dataset because this position is well-supported by the all-gene dataset and by the phylogeny presented by Deuve et al. [17]. Nucleotide substitution models were inferred during the MCMC analysis using the bModelTest package [74] implemented in BEAST2. Otherwise, we followed the settings used by Andújar et al. [16] and used a Yule process as a model of speciation, a strict molecular clock, and a random tree as a starting tree. Each run was performed with 100 million generations, sampling 10,000 trees and with a burn-in rate set to 10% of the samples. Convergence and stationary levels were verified using Tracer v1.7.1 [75]. We annotated the tree information using TreeAnnotator v.2.5.2 and visualized it with FigTree v.1.4.2. [76].

### Calibration analyses of each marker and their combinations

The analyses of time-calibrated phylogenies employing nodes A–C generally followed the protocol of Andújar et al. [16]. The median age and 95% height posterior densities (HPD) interval for the three clades were taken from the calibration analyses of the nd5 gene with BEAST2 (version 2.5.2) extracted with Tracer v 1.71. The gamma distribution was derived with the fitdistr function from the R package MASS (Shape 80.576, Scale 0.231; Shape 97.64, Scale 0.243; Shape 89.268, Scale 0.239 for nodes A, B, and C, respectively). We then calculated time-calibrated phylogenetic reconstructions of each gene, as well as the combinations of all mitochondrial genes, nuclear genes, and all mitochondrial and nuclear genes based on a run of 50 million generations. Subsequent steps followed the same protocol as in 3.3 (see above). The mean, standard error, highest posterior density intervals (HPD 95%), effective sample size of likelihood, evolutionary rates, and the TMRCA of *Carabus* were inspected using Tracer 1.7.1. Consensus trees were obtained in TreeAnnotator 2.5.2 [71] using the median age option. In all instances, an uncorrelated log-normal (ULN) relaxed clock was employed. All mitochondrial data sets were analyzed under a 2P codon partition scheme with site models and clock models unlinked.

## Results

### Calibration analyses with the extended nd5 data set

**Fig 1:**
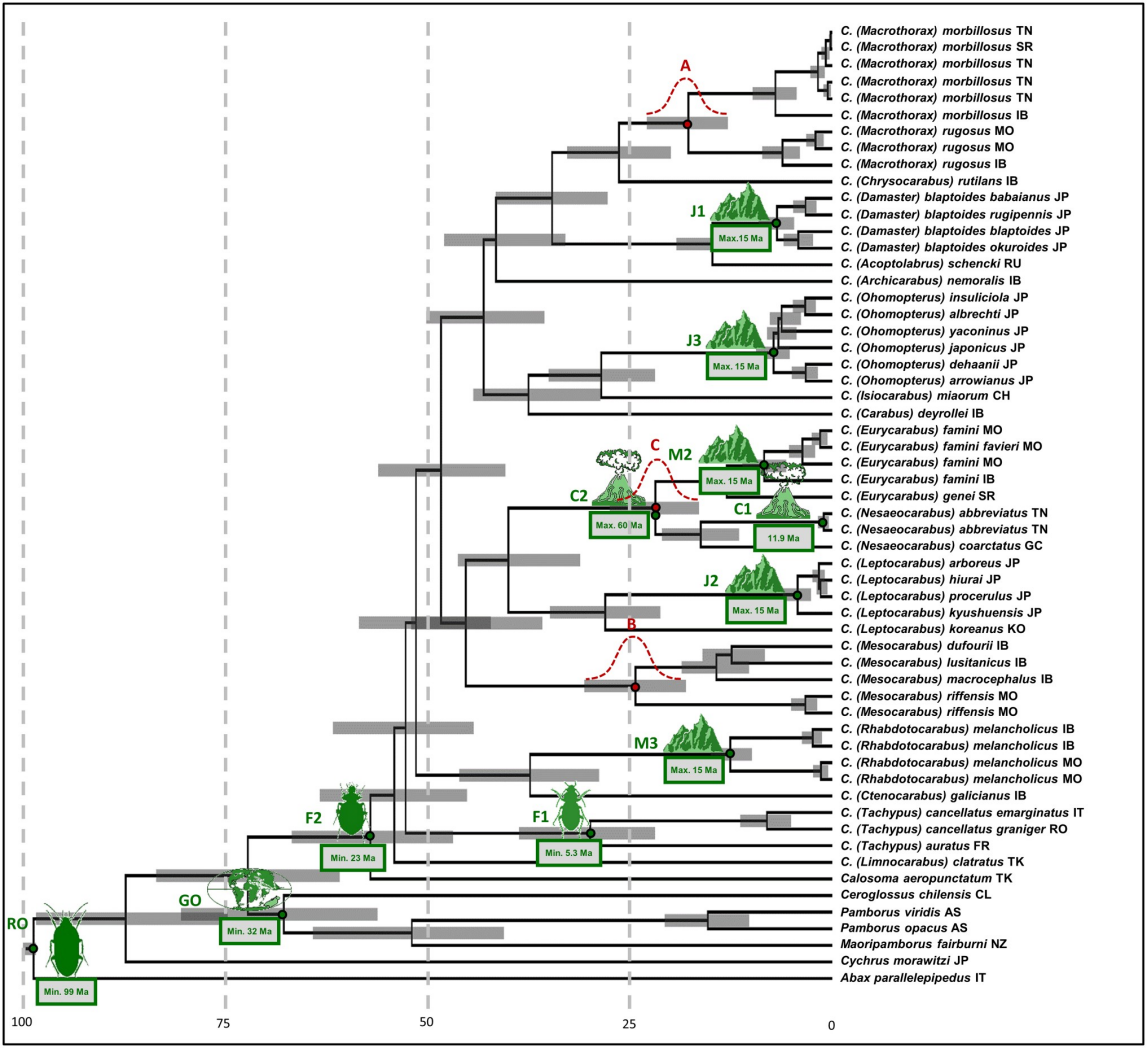
Ultrametric time-calibrated phylogenetic tree obtained with BEAST2 for nd5 data in *Carabus*. Green symbols depict calibration points employed (see Table 2 for details). Red symbols depict cladogenetic events whose age distributions (see Table 3) were utilized in subsequent analyses.

The expanded nd5 gene dataset of Andújar et al. [16] was used for our calibration analyses. A strict clock and 2P codon partitioning were implemented. The ultrametric time-calibrated phylogenetic tree is shown in Fig 1 with a median TMRCA *Carabus* of 53.92 Ma (HPD 95% 45.01–63.18 Ma), which was markedly older than the age obtained by Andújar et al. [16]. Nodes A, B, and C (Fig 1) had a median age of 17.58 (12.87–22.85), 24.14 (18.02–30.58), and 21.6 (16.44–27.43) Ma, respectively. They were more than twice as old as the values reported by Andújar et al. [16] (7.48 Ma, 10.93 Ma, and 9.51 Ma, see Table 1).

**Fig 2:**
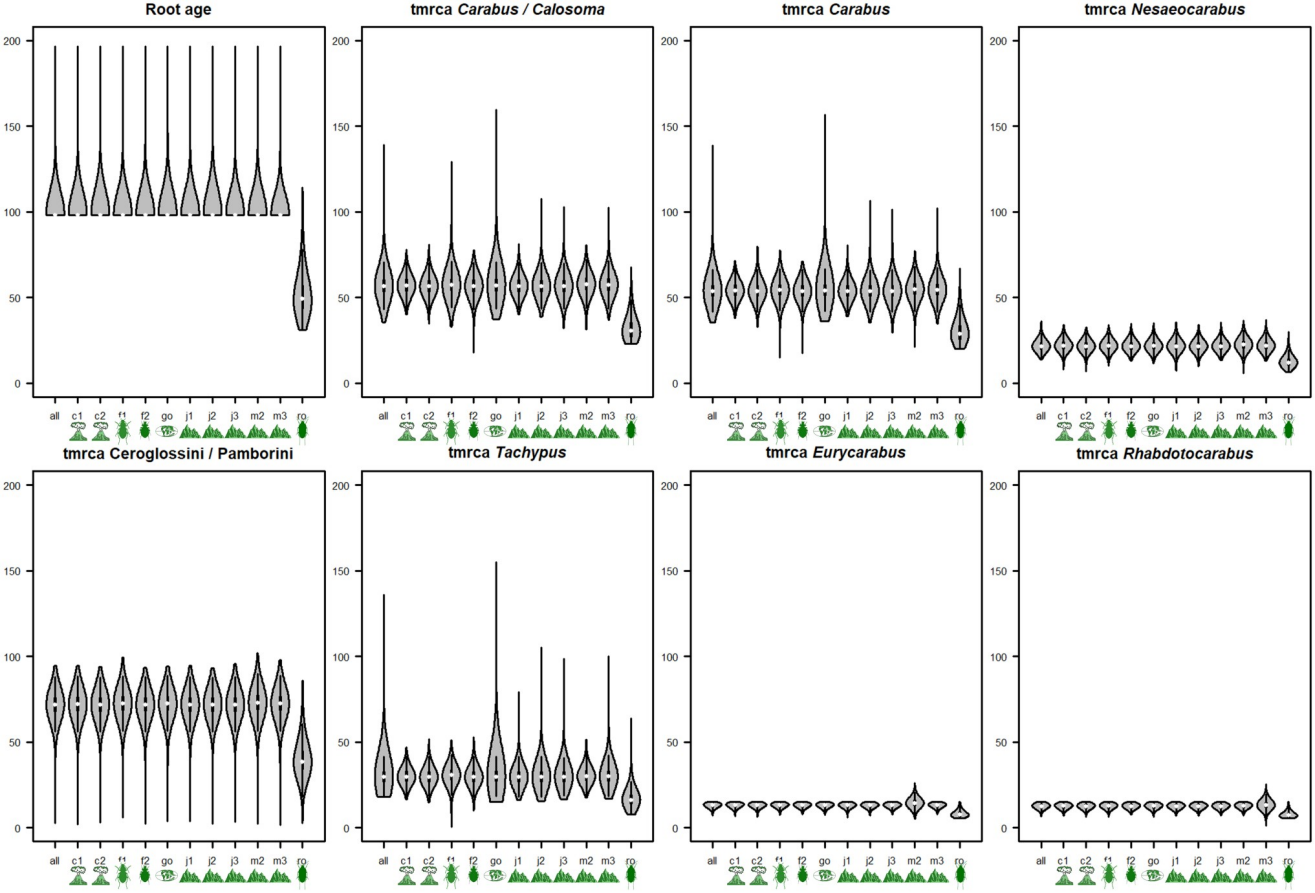
Violin plots of different TMRCA in relation to individual calibration points. The plots depict the mean (white dots), standard deviation (sd; black bars), 2*sd (black line), and density distribution (grey shape) of a clade’s age as obtained from the calibration analyses in Fig 1 for each major clade in relation to exclusion of individual calibration points. The x-axis denotes (letter) and depicts (icon) the respectively excluded calibration points in the respective analysis.

The violin plots in Fig 2 illustrate the overall impact of each calibration point on the timing of important phylogenetic lineages by depicting the age distribution when all points were used (all) and when each respective calibration point was left out of the calculation (-c1 through -root). As expected, this difference was mostly attributed to the root prior, which effectively overwrote any influence from other nodes, as evident by the limited impact of their omission on the dating results (Fig 2). In Table 3, this decisive impact is highlighted for clades A, B, and C, which are only 0.81, 0.69, and 1.21 Ma older than those in Andújar et al. [16] when the root prior was omitted. This is also reflected in the nearly similar lower bounds of the 95% height posterior densities (HPD). However, the upper bounds of the 95% HPD differ substantially between the two dating approaches. Importantly, while there is no overlap between the 95% HPD of the Andujar study and our root-calibration results, there is overlap for all three clades between both of our dating approach.

**Table 3:** Comparison of secondary calibration points derived in this study and in Andújar et al. [16] to calibrate the molecular phylogenies of the single (left) and combined datasets (right) in *Carabus*.

NODE	CLADOGENETIC EVENT	Andújar et al. [16] (Ma)	Without root calibration (Ma)	With all calibrations (Ma)
**A**	Split between *Carabus rugosus* and *C*. *morbillosus*	7.48	9.56	17.58
		(6.05–9.14)	(6.14–15.07)	(12.87–22.85)
**B**	Split of *Carabus riffensis* from European *Mesocarabus*	10.93	13.08	24.14
		(8.90–13.26)	(8.42–20.26)	(18.02–30.58)
**C**	Split between *Eurycarabus* and *Nesaeocarabus*	9.51	12.07	21.60
		(7.71–11.56)	(8.04–18.69)	(16.44–27.43)

Median age and 95% HPD interval for the three clades were taken from the calibration analyses of the nd5 gene with BEAST2 (version 2.5.2) extracted with Tracer v1.71. Gamma distribution was derived with the fitdistr function from the R package MASS. Four different sets of calibration points are shown in order of increasing age of the three clades: Without the root and Gondwana prior, as in Andújar et al. [16] without root calibration and with all calibrations.

**Fig 3:**
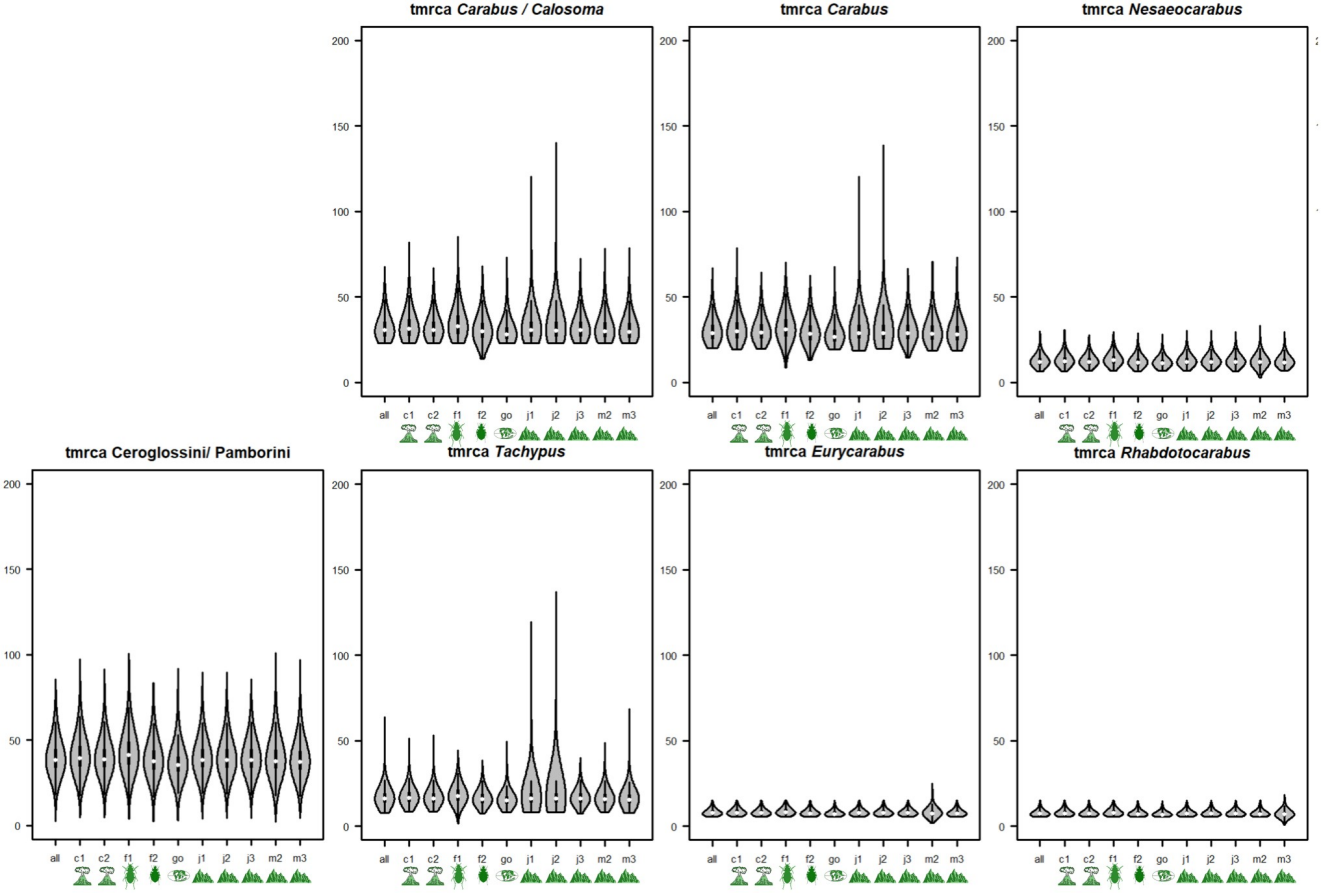
Violin plots of different TMRCA in relation to individual calibration points when root dating is omitted. The plots depict mean (white dots), standard deviation (sd; black bars), 2*sd (black line), and density distribution (grey shape) of a clade’s age as obtained from the calibration analyses in Fig 1 for each major clade in relation to exclusion of individual calibration points. The x-axis denotes (letter) and depicts (icon) the respectively excluded calibration points in the respective analysis.

The violin plots in Fig 3 are presented entirely without the root prior to highlight the impact of other calibration points. No other calibration point had such a decisive impact as the root did. However, as expected, fossils F1 and F2 invoked their minimum bound on all other priors in the *Carabus-Calosoma* split as well as the *Carabus* lineage (except for J3 for *Carabus*).

Expectedly, the recovery of nodes generally had similar posterior probabilities (PPs), as described by Andújar et al. [16]. Thus, nodes A, B, and C had a PP of 1.0 in all instances. Lineages A, B, and C were resolved in all-gene trees and gene-combination trees, except for cox1-a, LSU-A, and LSU-B. In the latter, none, A and C, and B and C, respectively, could be resolved with adequate PPs. The derived median TMRCA of *Carabus* varied markedly among genes ranging from 32.28 Ma for LSU-A to 84.17 Ma for ITS2. All medians and 95% HPD intervals for the respective genes and gene sets are given in Table 4. The ultrametric time-calibrated tree of the MIT-NUC data set led to a TMRCA of *Carabus* of 48.91 (38.33–60.95) Ma compared to the value of 25.16 (18.41–33.04) reported by Andújar et al. [16].

**Table 4:** Estimates of the molecular age of the MRCA of *Carabus* according to gene or gene set.

	This study			Andújar et al. [16]		
Gene (set)	Partition/ Clock	Median / mean age (Ma)	95% HPD interval (Ma)	Partition/ Clock	Mean age (Ma)	95% HPD interval (Ma)
***cox1-A***	2P/ULN	47.38 / 47.98	36.61–60.17	2P/SC	19.79	15.20–24.90
***cox1-B***	2P/ULN	46.01 / 46.53	35.27–58.38	2P/SC	21.58	16.43–27.71
***cytb***	2P/ULN	60.97 / 61.89	45.18–80.55	2P/SC	25.77	19.74–32.91
***nd5***	2P/ULN	48.88 / 49.40	37.67–61.68	NP/SC	20.71	151.9–26.15
***rrNL***	2P/ULN	58.34 / 59.86	38.24–83.84	NP/SC	29.91	19.40–42.76
***LSU-A***	NP/ULN	32.28 / 35.24	19.29–58.94	NP/ULN	13.37	8.35–24.85
***LSU-B***	NP/ULN	55.03 / 59.28	28.82–98.08	NP/ULN	20.36	11.23–36.61
***ITS2***	NP/ULN	84.17 / 88.51	45.35–140.24	NP/ULN	31.17	16.80–52.47
***HUWE1***	NP/ULN	62.35 / 64.15	40.11–91.81	NP/SC	30.83	21.79–41.36
***MIT***	2P/ULN	45.10 / 45.37	37.57–53.80	G-2P/SC	21.58	17.98–25.40
***NUC***	NP/ULN	66.46 / 69.14	39.91–104.42	NP/ULN	28.50	16.97–44.65
***MIT-NUC***	G-2P/ULN	48.91 / 49.40	38.33–60.95	G-2P/ULN	25.16	18.41–33.04

Estimates were obtained from an MCMC run of 50 million iterations (sampling parameter values and trees every 10,000 iterations, with a burn-in of 10%). Except for the evolutionary model used, which was inferred by bModelTest during the run, all parameters were the same for all analyses.

As mentioned in the Introduction, we did not focus on evolutionary rates. However, for comparability and potential future discussions we have provided evolutionary rates in S1 Table; rates are 1–2 orders of magnitude lower than reported in the Andújar study.

## Discussion

### Time scale of *Carabus* evolution

**Fig 4:**
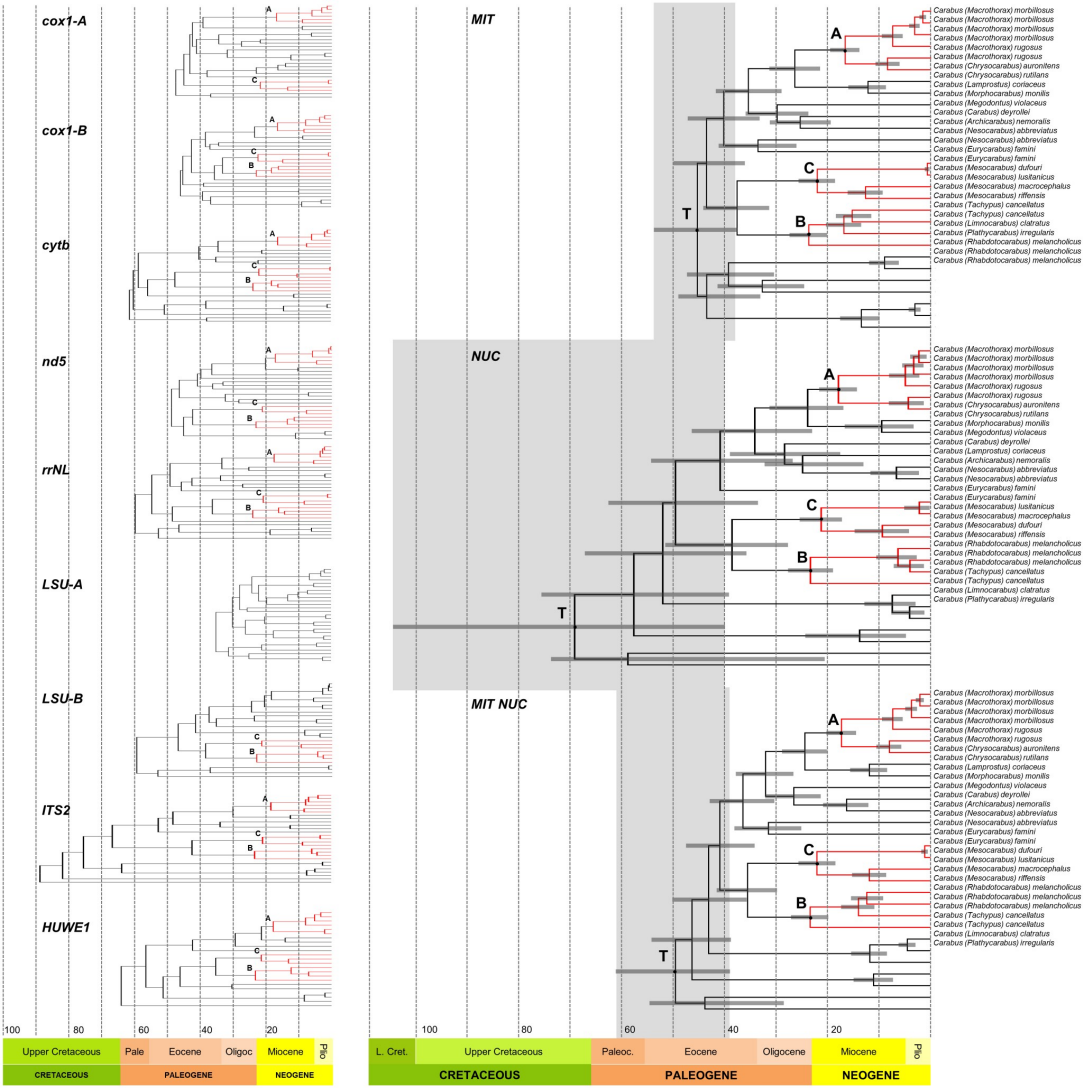
Ultrametric time-calibrated trees for combined DNA markers (MIT-NUC dataset) of Carabidae in this study (A) and Andújar et al. [16] (B). Analyses were conducted with BEAST2 and followed the approach of Andújar et al. [16] applying an uncorrelated log-normal relaxed clock. All mitochondrial data sets were included under a 2P codon partition scheme with site models and clock models unlinked. Posterior probabilities are given. Grey bars represent the 95% confidence intervals for node ages in Ma. The vertical brown bar shows the 95% HPD interval for the split between *Carabus* and *Calosoma*, while the vertical grey bar in the inset represents the same in the study of Andújar and coworkers.

We reanalyzed the data presented by Andújar et al. [16] on the phylogenetic timing of the genus *Carabus* in light of fossil evidence and the Gondwana split and offered a differing biogeographical interpretation of the geological record. We based our differing interpretation on a review of recent literature dealing with i) Taxa that have a Gondwanan distribution, extracting minimum and maximum calibration ages, ii) The onset of the Canary Hotspot, iii) The uplift of the western Mediterranean mountain ranges, and iv) The terrestrial evolution of the Japanese island arc in its orogenetic context. As expected, our study pushed the date of crown *Carabus* well beyond the Oligocene–Miocene dating of Andújar et al. [16] to the Eocene considering mitochondrial genes, and to the Cretaceous/Paleogene boundary considering the nuclear genes. Consequently, our findings support the models of Penev et al. [26] and Toussaint & Gillett [25] that posited the presence of *Carabus* species in the early Cenozoic. In addition, our timing does not support evidence for a phylogenetic standstill of 50–60 million years within Carabitae, as was assumed based on the data presented by Andújar et al. [16] (Fig 4). Furthermore, the Carabitae results are now also consistent with results from other taxa. One example is the dating of the Pelodryadinae-Phyllomedusinae split at 51.4 Ma (36.4–65.8) linked to the breakaway of Australia from South America [13]. We consider this resemblance significant as Anura show congruent evolutionary patterns with ground beetles based on similarly strong habitat ties [77, 78]Thus, this is likely not just a random match. However, we stress that the rewinding effect is almost entirely based on the fossils used to calibrate the root. Omitting these fossils led to rewinding of the clock of only roughly 1.5 million years (Table 3). Importantly though, when comparing the 95% height posterior densities, the values for our two approaches overlap while there is no overlap between the 95% HPD of the Andujar study and our root-calibration results. Thus, our treatment of the internal calibration points leads to results that are consistent with the approach including the root, while Andujar’s approach and the root-calibrated approach exclude each other and thus fail to reconcile the *Carabus* time-tree with the existence of the fossil record. Of the other calibration points, the strongest impact on the dating of the *Calosoma*-*Carabus* split and the *Carabus* ingroup could be attributed to ingroup fossils F2, and F1 and F2, respectively (see Fig 3).

**Fig 5:**
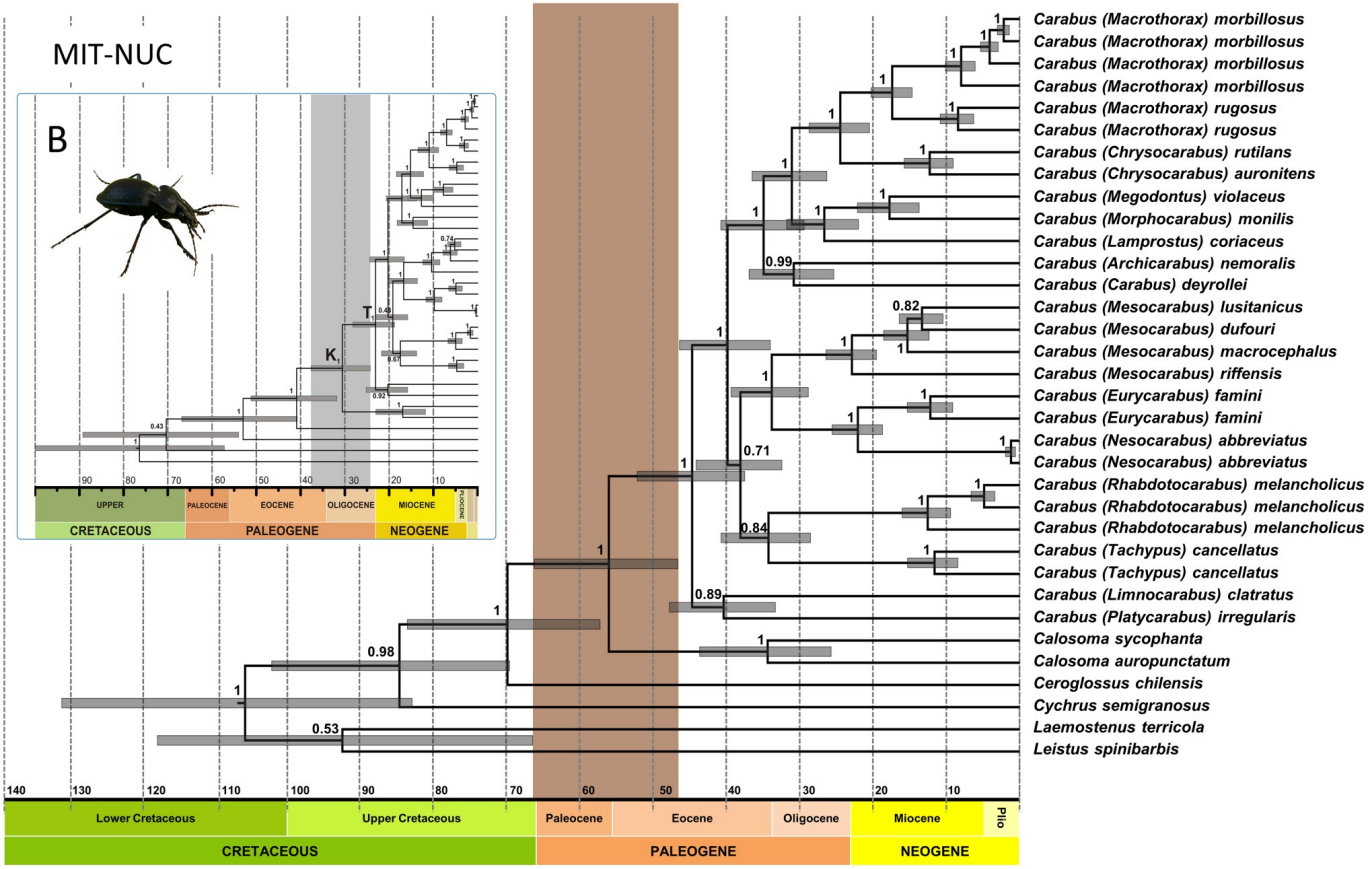
Ultrametric time-calibrated trees obtained with BEAST2 for each individual (left) and combined datasets (right) of the *Carabus* ingroup data set. Red clades and letters A, B, and C, are given for the individual gene trees when clades were supported by PPs of more than 0.8, and for the latter for the concatenated data sets. They are equivalent to the dated clades given in Table 3. Grey bars indicated the 95% HPD for the respective nodes, with the TMRCA bar being superposed for the respective phylogeny. T denotes the TMRCA of *Carabus*.

The impact of the Harpalinae fossils from Burmese amber as the root calibration had the greatest impact on the dating of the root itself, given that we implemented these fossils with a hard lower bound. In the extended nd5 calibration analyses, this translated to a difference of almost 50 million years for the root when this calibration point was included or left out (see Fig 2 and Table 2). Concerning the younger lineages, the root and other calibration points produced more congruent results (Fig 2). The pattern has a similar, but weaker, trend when comparing the ultrametric time-calibrated trees obtained from the MIT data and the NUC or the combined MIT/NUC dataset (Fig 5). This also implies that dating of the internal clades will likely not be pushed back much further unless additional older fossil evidence will appear. The initial split between *Carabus* and *Calosoma* occurred between and 47–67 Ma (Andújar et al., 33–18 Ma; [16]. This time was congruent with the final major phase of the breakup of Laurasia and opening of the northern Atlantic Ocean, and with the split of the Nearctic and Palearctic regions in this part of the northern hemisphere [79]. Andújar et al. [16] argued that the much younger *Carabus*/*Calosoma* split they inferred was congruent with the greater diversity of *Carabus* in the Palearctic region, particularly because of the low dispersal ability of flightless species. However, the assumption that the genus *Carabus* is flightless is misleading. Functional hind wings are occasionally present in the extant species *Carabus* (*Limnocarabus*) *clatratus*, particularly in *C*. (*Carabus*) *granulatus* of the more terminal Digitulati group. Thus, the capability of flight must have been a trait not only for the MRCA of the genus *Carabus* but for all MRCA of its major lineages. Consequently, flight has to be assumed as an important precondition for the colonization of several marginal parts of the genus’ area of distribution. This is particularly true in south locales, such as the Canary Islands and the North African Mountains, as discussed in the Materials and Methods section. Also, it is very likely a precondition for the trans-Palearctic and trans-Holarctic distributions in several of the extant lineages (apart from those species that were dispersed more recently by human activities). The evolutionary events that originated the main extant lineages, according to our data, took place during the Mid and Late Paleogene. This is much earlier than estimated by Andújar et al. [16] and Deuve et al. [17]. As such, they are probably associated with the reorganization of the terrestrial biomes of the northern hemispheric regions due to climatic shifts [55] and major geomorphological events in Central and East Asia resulting from the uplift of the Himalaya-Tibet orogenic system [80–83]. Since *Carabus* beetles are strictly adapted to temperate climates and are absent in the tropics, climatic shifts might have had major impacts on the early distributional history of the genus, while the Neogene orogenetic evolution of the northern hemisphere was the main driver for allopatric diversification within the terminal lineages. This resulted in an enormous number of wingless local endemic species, particularly in the mid-latitude mountains.

Given the considerable impact of root calibration on the dating results, future addition of Carabitae ingroup fossils originating from different geological periods to the analysis is mandatory. However, apart from *Calosoma* preserved in Late Oligocene deposits, pre-Miocene Carabitae fossils have not yet been found. Concerning the markedly diverging estimations of *Carabus-Calosoma* divergence time based on more comprehensive molecular phylogenetic beetle studies of approximately 133 Ma (98–172) by Toussaint & Gillet [25] and 34 Ma (18–57) by McKenna et al. [32] based on a large set of outgroup fossils, our results represent an intermediate position that may persist.

The same may hold true for the estimated evolutionary rates. However, we caution that due to the impact of the root calibration the saturation effect will likely affect the rate estimation strongly. Consequently, the evolutionary rates here derived, which are 1–2 orders of magnitude lower than in the study of Andújar, will likely lead to an overestimation of young nodes if applied to other phylogenies. As mentioned above, our goal in this study was not to discuss these rates in depth.

### Biogeographic dating in light of specific life-history

Preferences for certain climatic conditions as well as dispersal ability of the species group must be considered when geographical events are used to date phylogenetic events. Since *Carabus* is an extratropical genus with all its species having strictly adapted to warm temperate or colder climates, there is no doubt about the origin of the genus *Carabus* in the northern parts of the earlier Palearctic region. However, to date, there is no clear evidence for a more detailed geographical origin. Previous molecular phylogenetic studies of the genus by Deuve et al. [17] showed the simultaneous appearance of western (Arcifera, *Tachypus*) and eastern Palearctic elements (Crenolimbi, Spinulati) at the base of the *Carabus* tree. Reconstructing the early distributional history of the genus is more difficult because active dispersal by flight has to be assumed for the ancestors of most of the extant clades, as discussed in the previous section. However, previous phylogenetic studies were probably biased by the author’s preoccupation with extant *Carabus* species being 99% wingless. Thus, the true historical dispersal ability of the species was likely underestimated. For example, the emergence of the subgenus *Nesaeocarabus* on the Canary Islands, which was a dating point in previous phylogenetic studies of the genus [16, 17], has to be considered in light of active (flight) dispersal from North Africa or southwestern Europe. Dispersal by flight is likewise the most probable explanation for trans-Mediterranean distributional patterns, as observed in the subgenera *Eurycarabus* and *Rhapdotocarabus*. However, previous authors likely assumed dispersal “on foot” because of the desiccation of the Strait of Gibraltar during the Messinian crisis approximately 6–5 Ma. Thus, a short period of terrestrial connection of the continents was considered a prerequisite of the dispersal of the respective lineages. In the Materials and Methods section, we summarized arguments to reject this scenario because it is in strong contrast to the habitat preferences of the species. If active dispersal by flight is considered when discussing the dispersal history of the genus or using vicariance in biogeographic dating, the emergence and distribution of humid temperate habitats should be considered a much more important factor than terrestrial pathways. Consequently, concerning the trans-Mediterranean distributional patterns in some of the *Carabus* lineages and the Japan–Asian mainland disjunct distributional patterns of other lineages, in pre-Pliocene times, the distribution of mountainous areas as the provider of humid temperate habitats becomes the key factor for the distribution of potential paleohabitats of the species.

## Conclusions

Considering future dating analyses of ground beetles, the present findings stress the general need to invest more work into improving fossil databases and correctly placing them in phylogenies as crucial primary calibration points. In the present dating of the genus *Carabus*, the inclusion of fossil evidence combined with taxon-specific biogeographic features resulted in a much earlier time to the most recent common ancestor of this clade. However, our conclusions should be considered preliminary due to the strong impact of root calibration on the dating results. Additional Carabitae ingroup fossils are needed to prove our hypothesis, particularly those from pre-Miocene deposits. Furthermore, we stress the need for a more differentiated and transparent usage of geological calibration points depending on life-history traits and habitat requirements of the taxa under study. Geological events need to be strictly interpreted from a biogeographic perspective, including taxon-specific habitat suitability and dispersal abilities. Geological events, such as the presence or interruption of land bridges caused by sea-level changes, are often transferred uncritically from a taxon where they might have enacted significant evolutionary pulses to taxa where they do not.

## Supporting information

S1 Table Comparison of rates of molecular evolution of *Carabus* for each individual fragment and combined datasets between this study and Andújar et al. [1]. The data is corresponding to the ingroup dataset. Rates are given in substitutions per site per million years per lineage. The rates are estimated after character culling in G-blocks and are therefore underestimated.
(DOCX)
