# Supplementary material for: Rewinding the molecular clock in the genus Carabus (Coleoptera: Carabidae) in light of fossil evidence and the Gondwana split: A reanalysis
Source: PLoS One. 2021 Sep 22;16(9):e0256679. doi: 10.1371/journal.pone.0256679 (PMC8457462; doi:10.1371/journal.pone.0256679)
Supplement: S1 Table — The data is corresponding to the ingroup dataset. Rates are given in substitutions per site per million years per lineage. The rates are estimated after character culling in G-blocks and are therefore underestimated. (DOCX) [file pone.0256679.s001.docx]

Table S1. Comparison of rates of molecular evolution of *Carabus* for each individual fragment and combined datasets between this study and Andújar et al. [1]. The data is corresponding to the ingroup dataset. Rates are given in substitutions per site per million years per lineage. The rates are estimated after character culling in G-blocks and are therefore underestimated.

|  | **This study** | | | **Andújar et al.** [16] | | |
| --- | --- | --- | --- | --- | --- | --- |
| **Gene (set)** | Partition/ Clock | Mean rate | 95 % HPD  interval | Partition/ Clock | Mean rate | 95 % HPD  interval |
| ***cox1-A*** | 2P/ULN | 0.0004 | 0.0003-0.0007 | 2P/SC | 0.0113 | 0.0081-0.0147 |
| ***cox1-B*** | 2P/ULN | 0.0006 | 0.0003-0.001 | 2P/SC | 0.0145 | 0.01-0.0198 |
| ***cytb*** | 2P/ULN | 0.0011 | 0.0006-0.0016 | 2P/SC | 0.0251 | 0.0151-0.0369 |
| ***nd5*** | 2P/ULN | 0.0006 | 0.0004-0.0009 | NP/SC | 0.0159 | 0.0102-0.0223 |
| ***rrNL*** | 2P/ULN | 0.0007 | 0.0004-0.0009 | NP/SC | 0.0016 | 0.001-0.0022 |
| ***LSU-A*** | NP/ULN | 0.0006 | 0.0003-0.0008 | NP/ULN | 0.0013 | 0.0007-0.002 |
| ***LSU-B*** | NP/ULN | 0.003 | 0.0018-0.0043 | NP/ULN | 0.0064 | 0.0037-0.0094 |
| ***ITS2*** | NP/ULN | 0.003 | 0.0016-0.0035 | NP/ULN | 0.0057 | 0.0035-0.0081 |
| ***HUWE1*** | NP/ULN | 0.0009 | 0.0007-0.0012 | NP/SC | 0.0021 | 0.0015-0.0027 |
| ***MIT*** | 2P/ULN | 0.004 | 0.0030-0.0044 | G-2P/SC | 0.0134 | 0.0108-0.0162 |
| ***NUC*** | NP/ULN | 0.001 | 0.0009-0.0016 | NP/ULN | 0.0029 | 0.002-0.0039 |
| ***MIT-NUC*** | G-2P/ULN | 0.002 | 0.0018-0.0025 | G-2P/ULN | 0.0080 | 0.0064-0.0097 |
